# Spread of Linezolid-Resistant *Enterococcus* spp. in Human Clinical Isolates in the Czech Republic

**DOI:** 10.3390/antibiotics10020219

**Published:** 2021-02-22

**Authors:** Lucia Mališová, Vladislav Jakubů, Katarína Pomorská, Martin Musílek, Helena Žemličková

**Affiliations:** 1 National Reference Laboratory for Antibiotics, Centre for Epidemiology and Microbiology, National Institute of Public Health, 10000 Prague, Czech Republic; lucia.malisova@szu.cz (L.M.); vladislav.jakubu@szu.cz (V.J.); katarina.pomorska@szu.cz (K.P.); 2 Department of Microbiology, 3rd Faculty of Medicine Charles University, University Hospital Kralovske Vinohrady and National Institute of Public Health, 10000 Prague, Czech Republic; 3 Department of Clinical Microbiology, Faculty of Medicine and University Hospital, Charles University, 53002 Hradec Kralove, Czech Republic; 4 National Reference Laboratory for Meningococcal Infections, Centre for Epidemiology and Microbiology, National Institute of Public Health, 10000 Prague, Czech Republic; martin.musilek@szu.cz

**Keywords:** *Enterococcus faecium*, *Enterococcus faecalis*, linezolid resistance, 23S rRNA, optrA

## Abstract

The aim of this study was to map and investigate linezolid resistance mechanisms in linezolid-resistant enterococci in the Czech Republic from 2009 to 2019. Altogether, 1442 isolates of *Enterococcus faecium* and *Enterococcus faecalis* were examined in the National Reference Laboratory for Antibiotics. Among them, 8% of isolates (*n* = 115) were resistant to linezolid (*E. faecium*/*n* = 106, *E. faecalis*/*n* = 9). Only three strains of *E. faecium* were resistant to tigecycline, 72.6% of isolates were resistant to vancomycin. One isolate of *E. faecium* harbored the *cfr* gene. The majority (87%, *n* = 11) of *E. faecium* strains were resistant to linezolid because of the mutation G2576T in the domain V of the 23S rRNA. This mutation was detected also in two strains of *E. faecalis*. The presence of the *optrA* gene was the dominant mechanism of linezolid resistance in *E. faecalis* isolates. None of enterococci contained *cfrB*, *poxtA* genes, or any amino acid mutation in genes encoding ribosomal proteins. No mechanism of resistance was identified in 4 out of 106 *E. faecium* linezolid resistant isolates in this study. Seventeen sequence types (STs) including four novel STs were identified in this work. Clonal complex CC17 was found in all *E. faecium* isolates.

## 1. Introduction

Enterococci are Gram-positive bacteria, commensals of the gastrointestinal tract and opportunistic pathogens able to cause community-acquired and nosocomial infections. Two species, *Enterococcus faecium* and *Enterococcus faecalis*, are considered to be one of the most important nosocomial pathogens worldwide [1]. They cause life-threatening infections especially in elderly, polymorbid and immunocompromised patients [2]. Increasing resistance of enterococci to penicillin, aminoglycosides, glycopeptides or to the last resort antibiotics (daptomycin, tigecycline, linezolid) prevents these drugs from being effective in the treatment of infections caused by these bacteria [3].

Linezolid is a bacteriostatic antibiotic efficient only against Gram-positive bacteria including methicillin-resistant *Staphylococcus aureus* (MRSA) and vancomycin resistant enterococci [4]. It inhibits the accuracy of the protein translation by binding to the peptidyl transferase centrum (PTC) in the V domain of the 23S rRNA inside the 50S ribosomal subunit [5]. Since the introduction of linezolid into clinical use in 2000 (USA) [4], seven mechanisms have been described as related to the linezolid resistance in enterococci: Mutations in the 23S rRNA and genes encoding ribosomal proteins L3, L4, and L22, the acquisition of plasmid carrying genes *cfr* (chloramphenicol-florfenicol) [6], *optrA* (ABC transporter that confers resistance to oxazolidinones and phenicols) [7] and *poxtA* (ABC transporter; resistance to oxazolidinones, phenicols and tetracyclines) [8]. The most common mechanism of linezolid resistance in enterococci is the conversion of G to T at position 2576 in the 23S rRNA [9]. Ribosomal proteins L3, L4, L22 play an important role in the stabilization and conformation of the ribosome (PTC). Therefore, mutations in genes (*rplC*, *rplD,* and *rplV*) encoding these proteins lead to the amino acid changes followed by disruption of translation. This type of mechanism is predominantly linked with linezolid resistance in *Staphylococcus epidermidis* [10].

The gene *cfr* encodes a methyltransferase that catalyzes the posttranscriptional methylation of nucleotide A2503 in the 23S rRNA [10]. It has been described for the first time in *S. aureus* in 2005 [11], and it can be transferred across different bacterial species and genera [12]. New mechanisms, *optrA* (China, 2015) [7] and *poxtA* [8], belonging to the ABC-F family of ATP-binding cassette (efflux pump genes) were revealed recently. Comparative analysis at the protein levels in the genome of linezolid resistant *S. aureus* revealed 32% protein homology between them [8]. Gene *optrA* has been more often detected in livestock than in humans and its occurrence is more associated with *E. faecalis* than *E. faecium* strains [1]. *PoxtA* was originally identified in Italian isolate MRSA in 2018 [8] and its prevalence amongst the enterococcal population is still under investigation.

The purpose of this study was to investigate the spread of linezolid resistant enterococci acquired from human clinical specimens in the Czech Republic over the period of 10 years, and to analyze molecular mechanisms of their resistance to linezolid.

## 2. Results

### 2.1. Antibiotic Susceptibility of Linezolid Resistant Isolates of E. faecium and E. faecalis

Altogether, 1442 enterococcal isolates (791/58.5%/*E. faecium*, 651/45%/*E. faecalis*) were examined in the National Reference Laboratory for Antibiotics from 2009 to 2019. Of them, 115 strains (8%) were resistant to linezolid: 106 isolates (13.4%) of *E. faecium* and 9 strains (1.4%) of *E. faecalis*. The number of linezolid resistant enterococci increased from 2009 to 2019 (*E. faecium*; from 2009/0 to 2019/32, *E. faecalis*; from 2009/0 to 2019/4, Supplementary Figure S1). Resistance to vancomycin was confirmed in 72.6% (*n* = 77) of *E. faecium* strains. Resistance to teicoplanin was detected in 70% (*n* = 74) of isolates, 2.8% (*n* = 3) of strains were resistant to tigecycline. The majority of linezolid resistant *E. faecium* isolates were resistant also to gentamicin (76.5%, *n* = 89) and streptomycin (69%, *n* = 79). None of linezolid resistant *E. faecalis* strains was resistant to ampicillin, teicoplanin, vancomycin and tigecycline. All of them were resistant to gentamicin, 4 isolates also to streptomycin (Table 1, Supplementary Table S1).

### 2.2. Mechanisms of Linezolid and Vancomycin Resistance

Altogether, 93.4% (*n* = 99) of *E. faecium* isolates harbored the point mutation G2576T in the V domain of the 23S rRNA, two strains of *E. faecium* were positive for the presence of *optrA* gene, one isolate was *cfr* positive. There was not revealed the mechanism of linezolid resistance in four isolates. In 7 out of 9 *E. faecalis* isolates, the presence of *optrA* gene was confirmed, the mutation G2576T was revealed in two samples. Resistance to vancomycin was detected only in *E. faecium* strains. The presence of *vanA* (92%, *n* = 71), and *vanB* (*n* = 2) genes was confirmed. Four isolates were resistant to vancomycin due to the combination of *vanA*, *vanB* (*n* = 4) genes (Table 1, Supplementary Table S1).

### 2.3. Molecular Typing-MLST Analysis

Altogether, 12 different sequence types (STs) were found in linezolid resistant *E. faecium* strains. The most frequent STs detected were ST80 (*n* = 53) and ST117 (*n* = 24), followed by ST18 (*n* = 13), ST761 (*n* = 4), ST78 (*n* = 3). Other STs were represented by a single or two isolates: ST17 (*n* = 2), ST203 (*n* = 1), ST552 (*n* = 1), ST262 (*n* = 1). Due to the new type of *gyd* allele, three novel STs were identified in the study: ST1487 (*n* = 1), ST3501 (*n* = 2), ST3502 (*n* = 1) (Supplementary Figure S2). Almost all strains of *E. faecium* (97%, *n* = 103) belonged to the same clonal complex, CC17 (Table 1, Supplementary Table S1). MLST typing revealed a high genetic variability within the group of *E. faecalis* strains. Altogether, there were confirmed 5 STs: ST6/CC2 (*n* = 1)/, ST476/CC476 (*n* = 3), ST480 (*n* = 2), ST858 (*n* = 2) and a new ST1982 (*n* = 1) (Supplementary Figure S3).

## 3. Discussion

This is the first study mapping linezolid resistant enterococci acquired from human clinical specimen in the Czech Republic. Enterococci have become one of the most prevalent (nosocomial) pathogens over the past decades and linezolid provides one of the therapeutic options for infections caused by this bacteria.

Among 1442 enterococci sent to the National Reference Laboratory for Antibiotics from 2009 to 2019, 8% of them were resistant to linezolid. Congruently with other European countries, the pattern of occurrence of linezolid resistant enterococci increased from year to year (0/2009-36/2019) [9,13]. Except for 3 strains (2.6%) almost all analyzed enterococci were susceptible to tigecycline, an alternative option in the treatment of enterococcal infections. In accordance with this observation, the majority of the European countries reports generally low prevalence (<1%) of isolates resistant to last-resort antibiotics (daptomycin, tigecycline, linezolid) [14]. A lower rate of linezolid resistance in enterococci could be explained by lower selection pressure of this antibiotic or by its mechanism. The substitution of G to T in the position 2576 in the 23S rRNA develops as a spontaneous mutation. It was reported that the frequency of the spontaneous resistance to linezolid (in enterococci) is lower than to other antibiotics [15]. This statement has been confirmed also in this work, 87.8% (101/115) of linezolid resistant enterococci contained the mutation G2576T in the 23S rRNA [16]. Mutations in *rplC*, *rplD*, and *rplV* genes were not observed in this work. Mutations in genes encoding ribosomal proteins (L3, L4, L22) were more commonly seen in coagulase-negative staphylococci (preferentially *S. epidermidis*) than in enterococci [17].

The rate of *E. faecium* isolates harboring gene *optrA* was negligible. Altogether, only two isolates (ST262, ST3502) were positive for the *optrA* gene. This result is not surprising, the occurrence of *optrA* has been associated more with *E. faecalis* than *E. faecium* species [18,19]. The presence of the gene *optrA* was the dominant mechanism responsible for the linezolid resistance in *E. faecalis* strains. It is worth noting, that *optrA* positive linezolid resistant enterococci can confer MICs of linezolid different than other linezolid resistant enterococci [20]. All enterococci positive for the gene *optrA* were associated with MICs for linezolid at the level up to 8 mg/L. The majority of enterococci harboring the G2576T mutation had MICs ≥ 16 mg/L.

None from enterococci analyzed in this work harbored the gene *poxtA*. This, a novel antibiotic resistance determinant, was until now identified only in one clinical isolate of *E. faecium* in Greece in 2018 [21]. Despite the sporadic occurrence of the *poxtA*, the scattered distribution of this gene among the different Gram-positive species (e.g., *S. aureus*, *Enterococcus* spp., etc.) deserves an attention [22].

At present, eight different types of *van* genes conferring vancomycin resistance mechanisms are known. The *vanA* and *vanB* resistance genotypes are the most frequently detected variants in clinical isolates of *E. faecium* and *E. faecalis* worldwide [14]. Vancomycin resistance caused by the presence of gene *vanA* was a dominant mechanism in the group of vancomycin resistant enterococci in the Czech Republic. Due to the location of the gene *vanA* on plasmid, this fragment of DNA can spread easier than *vanB*, which is usually a part of the bacterial chromosome [23]. Based on the global spread of enterococci resistant to the different kinds of antibiotics (including the last resort antibiotics), a molecular epidemiology studies are performed to obtain insights into the dissemination of these strains. MLST is a molecular typing method with suitable discriminatory power [23] very often used for this purpose. One pandemic clone of CC17 was detected in linezolid resistant *E. faecium* isolates in the Czech Republic from 2009 to 2019. The enterococcal lineage of CC17 is responsible for the spread of linezolid and vancomycin resistance in hospitals all around the world [1]. Moreover, strains belonging to this clonal complex show persistence in the environment and high colonization capability [24]. In this study, linezolid resistant enterococci belonged to 12 different STs, but a majority (50%) of isolates typed as ST80 was observed [25]. ST80 was described for the first time in blood of Israeli patient in 1997 (unpublished data, the source: pubmlst.org). Since then it has been detected all over the world. ST18, ST78, and ST117 were associated with enterococci also in other European countries [24,25]. ST17, ST203, and ST552 were observed earlier by Egan et al. in Ireland [26]. In concordance with others, a high risk STs associated with linezolid resistance in enterococci involved also ST262 [27], ST761 [28] and newly identified STs 3501, 3502, 1487 and 1982. A classical hospital-associated CC2 (ST6) of *E. faecalis* has been already detected in Spain and Poland [29,30]. STs 585 and 476 have been observed in overall diverse population of *optrA*-positive *E. faecalis* strains in Portugal [31].

Inability to infer the linezolid resistance mechanism in three *E. faecium* isolates suggests the possibility of presence of additional mechanisms of resistance. It is supported by results of studies on enterococci with a silent mechanism of resistance, but still exhibiting linezolid resistance [32]. Enterococci are adaptable bacteria characterized by a high plasticity of genome (a high rate of DNA recombination). Therefore, novel mechanisms of resistance to different kinds of antibiotics have emerged relatively rapidly. Efflux pumps, cell wall thickness and biofilm formation are still discussed as putative alternative pathways of linezolid resistance [33,34].

In conclusion, this study provided the first insight into the population structure of linezolid resistant enterococci in the Czech Republic within the period of 10 years. It showed that the rate of linezolid resistant enterococci was comparable with other European countries and it increased in both groups of examined enterococci. The main mechanism of linezolid resistance among clinical *E. faecium* isolates was the G2576T mutation in the domain V of the 23S rRNA. The presence of gene *optrA* was the major cause of linezolid resistance in *E. faecalis* strains. A high risk clone CC17 was the only CC detected in linezolid resistant *E. faecium* strains in the Czech Republic within last decade.

Still increasing prevalence of enterococci resistant to the last resort antibiotics as well as their ability to acquire novel DNA fragments encoding (new) resistance determinants predestine these bacteria to even more successful spreading. Therefore, enterococci resistant to linezolid represents a public health concern and monitoring the spread of these bacteria is necessary.

## 4. Materials and Methods

### 4.1. Bacterial Isolates

Screening of *E. faecium*/*E. faecalis* strains is performed as a part of European Antimicrobial Resistance Surveillance Network (EARS-Net) and the study of Monitoring of Antibiotic Resistance in the National Reference Laboratory for Antibiotics (National Institute of Public Health, Prague, Czech Republic). Enterococci presented in this study were acquired from 40 laboratories in the period from 2009 to 2019. The majority of strains, 61% (*n* = 875), was of invasive origin (blood; *n* = 873, cerebrospinal fluid; *n* = 2). The rest of isolates was acquired form non-invasive clinical specimens, and involved surgical wound, urine, pus, sputum, catheter, aspirate, bile, and swab (mouth, throat, nose, and vagina). Altogether, 5% (*n* = 70) of enterococci were isolated from rectal swab and stool sample. One isolate was of unknown origin. Characteristics of linezolid resistant strains are given in the Supplementary Table S1. Enterococci resistant to linezolid are further examined to reveal the mechanism of linezolid resistance and their epidemiological relationship (MLST). Resistance to linezolid (≥4 mg/L) was defined according to the European Committee on Antimicrobial Susceptibility Testing (EUCAST) breakpoint (www.eucast.org). All strains were routinely cultivated on Columbia blood agar (Oxoid, Brno, Czech Republic) aerobically at 36 ± 1 °C. Identification of strains was performed by Matrix-Assisted Laser Desorption Ionization-Time of Flight Mass Spectrometry (MALDI-TOF MS; Microflex Bruker, Bremen, Germany) according to the manufacturer’s protocol (www.bruker.com).

### 4.2. Susceptibility Testing

Minimal inhibitory concentrations (MICs) of ampicillin, linezolid, teicoplanin, vancomycin, gentamicin, streptomycin, and tigecycline were determined by broth microdilution method according to ISO 20776-1. Interpretation of susceptibility testing results was performed as recommended by EUCAST, version according to a corresponding year (last used version 9.0). Strains ATCC 29212 and ATCC 51299 were used as quality control in this study (both strains recommended by EUCAST; www.eucast.org).

### 4.3. Detection of Determinants of Linezolid Resistance

Mechanism of resistance to linezolid was determined by PCR (*cfr*, *cfrB*, *oprA*, and *poxtA*) and Sanger sequencing (23S rRNA, *rplC*, *rplD*, and *rplV*). DNA extraction was obtained from a fresh culture (24 hours) according to the manufacturer’s protocol (GenElute TM Bacterial Genomic DNA Kits, Sigma Aldrich, St. Louis, MO, USA). The list of enterococcal specific primers and settings of PCR used in the study are given in the Supplementary Table S2. PCR products of *cfr*, *oprA* were resolved in 1.5% agarose (TopVision Agarose, Thermo Scientific, St. Louis, MO, USA) in electrophoresis (5 V/cm) for 45 minutes. NCTC13923 was used as control strain for *optrA* detection in this study. The detection of *cfr*, *poxtA*, and *cfrB* was performed according to procedures as described previously [8,12,35]. The amplified fragments of the 23S rRNA, *rplC*, *rplD*, and *rplV* were sequenced by analyzer Applied Biosystems 3130xL. Point mutation/s associated with linezolid resistance were analyzed using software Bionumerics 7.6.2 (Applied Maths, Ghent, East Flanders, Belgium).

### 4.4. Detection of Mechanism of Vancomycin Resistance

Isolates resistant to linezolid and simultaneously resistant to vancomycin were further examined. The mechanism of resistance to vancomycin was screened by PCR using primers under conditions that are listed in the Supplementary Table S2. Electrophoresis was carried out as described above.

### 4.5. MLST Typing of Linezolid Resistant Strains of E. faecium, E. faecalis

Epidemiology of enterococci was investigated by the multilocus sequence typing (MLST) analysis as described earlier [36]. Seven primers targeting *adk* (adenylate kinase), *atpA* (ATP synthase, alpha subunit), *ddl* (d-alanine:d-alanine ligase), *gyd* (glyceraldehyde-3-phosphate dehydrogenase), *gdh* (glucose-6-phosphate dehydrogenase), *purK* (phosphoribosylaminoimidazol carboxylase ATPase subunit) and *pstS* (phosphate ATP-binding cassette transporter) alleles were used to amplify target region in *E. faecium* isolates. *PstS* (phosphate ATP binding cassette transporter), *gki* (putative glucokinase), *aroE* (shikimate 5-dehydrogenase), *xpt* (shikimate 5-dehydrogenase), *gyd*, *gdh,* and *yiqL* (acetyl-coenzyme A acetyltransferase) were used for MLST analysis of *E. faecalis* strains. Alleles *gyd* and *gdh* were amplified using the same primers for both species. The list of primers used for MLST analysis of enterococci is given in the Supplementary Table S3. All sequences were processed by analyser (Applied Biosystems 3130xL, Foster City, CA, USA). Allelic profiles, sequence types (STs) and clonal complexes (CC) of enterococci were determined using Bionumerics 7.6.2 (Applied Maths, Ghent, East Flanders, Belgium) and free available website pubmlst.org (https://pubmlst.org/organisms).

## Figures and Tables

**Table 1:** Molecular characteristics and antibiotic susceptibility of 115 human clinical isolates of *Enterococcus faecium* (*n* = 106) and *Enterococcus faecalis* (*n* = 9) analyzed in the study. Absolute numbers are expressed in % (number of isolates) and depict the number of resistant population in the group.

	E. faecium												
ST	CC	Number of Isolates	AMP	LNZ	TEI	VAN	GEN	STR	TGC	Mechanism of LNZ-R		Van Genotype	
**ST80**	17	53	100(53)	100(53)	94 (50)	94(50)	98(52)	85(45)	5.6 (3)	∆G2576T	(53)	*vanA*	(50)
**ST117**	17	24	100(24)	100(24)	45.8 (11)	54(13)	75(18)	83(20)	0	∆G2576T	(22)	*vanA*	(10)
										*cfr*	(1)	*vanB*	(2)
										*	(1)	*vanA,vanB*	(1)
**ST18**	17	13	100(13)	100(13)	46(6)	53.8(7)	84.6(11)	38.4(5)	0	∆G2576T	(12)	*vanA*	(5)
										*	(1)	*vanA,vanB*	(2)
**ST761**	17	4	100(4)	100(4)	25(1)	25(1)	75(3)	75(3)	0	∆G2576T	(3)	*vanA*	(1)
										*cfrB*	(1)		
**ST78**	17	3	100(3)	100(3)	33.3(1)	33.3(1)	33.3(1)	66.6(2)	0	∆G2576T	(3)	*vanA,vanB*	(1)
**ST17**	17	2	100(2)	100(2)	50(1)	50(1)	0	0	0	∆G2576T	(2)	*vanA*	(1)
**ST203**	17	1	100(1)	100(1)	100(1)	100(1)	0	100(1)	0	∆G2576T	(1)	*vanA*	(1)
**ST552**	17	1	100(1)	100(1)	0	0	100(1)	0	0	∆G2576T	(1)	-	
**ST262**	17	1	100(1)	100(1)	0	0	0	100(1)	0	*optrA*	(1)	-	
**ST1487 †**	17	1	100(1)	100(1)	100(1)	100(1)	100(1)	100(1)	0	*	(1)	*vanA*	(1)
**ST3501 †**	17	2	100(2)	100(2)	100(2)	100(2)	100(2)	0	0	∆G2576T	(2)	*vanA*	(2)
**ST3502 †**	17	1	0	100(1)	0	0	0	100(1)	0	*optrA*	(1)	-	
	*E. faecalis*												
**ST6**	2	1	0	100(1)	0	0	100(1)	100(1)	0	∆G2576T	(1)	-	
**ST476**	476	3	0	100(3)	0	0	100(3)	0	0	*optrA*	(3)	-	
**ST480**	480	2	0	100(2)	0	0	100(2)	50(1)	0	*optrA*	(2)	-	
**ST858**	unknown	2	0	100(2)	0	0	100(2)	100(2)	0	*optrA*	(2)	-	
**ST1982 †**	unknown	1	0	100(1)	0	0	100(1)	0	0	∆G2576T	(1)	-	

ST, sequence type; CC, clonal complex; AMP, ampicillin; LNZ, linezolid; TEI, teicoplanin; VAN, vancomycin; GEN, gentamicin; STR, streptomycin; TGC, tigecycline; *, unknown mechanism of linezolid resistance; LNZ-R, linezolid resistance; -, wild type; †, novel sequence types.

## Data Availability

The data presented in this study are available on request from the corresponding author.

## Supplementary Materials

The following are available online at https://www.mdpi.com/2079-6382/10/2/219/s1, Figure S1: Detection of linezolid resistant enterococci acquired from human clinical specimen in the Czech Republic from 2009 to 2019, Figure S2: STs occurrence in the group of linezolid resistant *E. faecium* (*n* = 106) strains from 2009 to 2019, Figure S3: STs occurrence in the group of linezolid resistant *E. faecalis* (*n* = 9) isolates from 2009 to 2019, Table S1: Phenotypic and genotypic characteristics of the 115 linezolid-resistant enterococcal isolates examined in the NRL for ATB between 2009 and 2019, Table S2: Primers used in the study, Table S3: Primers used in the MLST analysis of *E. faecium* and *E. faecalis* isolates.
