# Supplementary material for: Spread of Linezolid-Resistant Enterococcus spp. in Human Clinical Isolates in the Czech Republic
Source: Antibiotics (Basel). 2021 Feb 22;10(2):219. doi: 10.3390/antibiotics10020219 (PMC7927076; doi:10.3390/antibiotics10020219)
Supplement: Supplementary file 1 [file antibiotics-10-00219-s001.pdf]

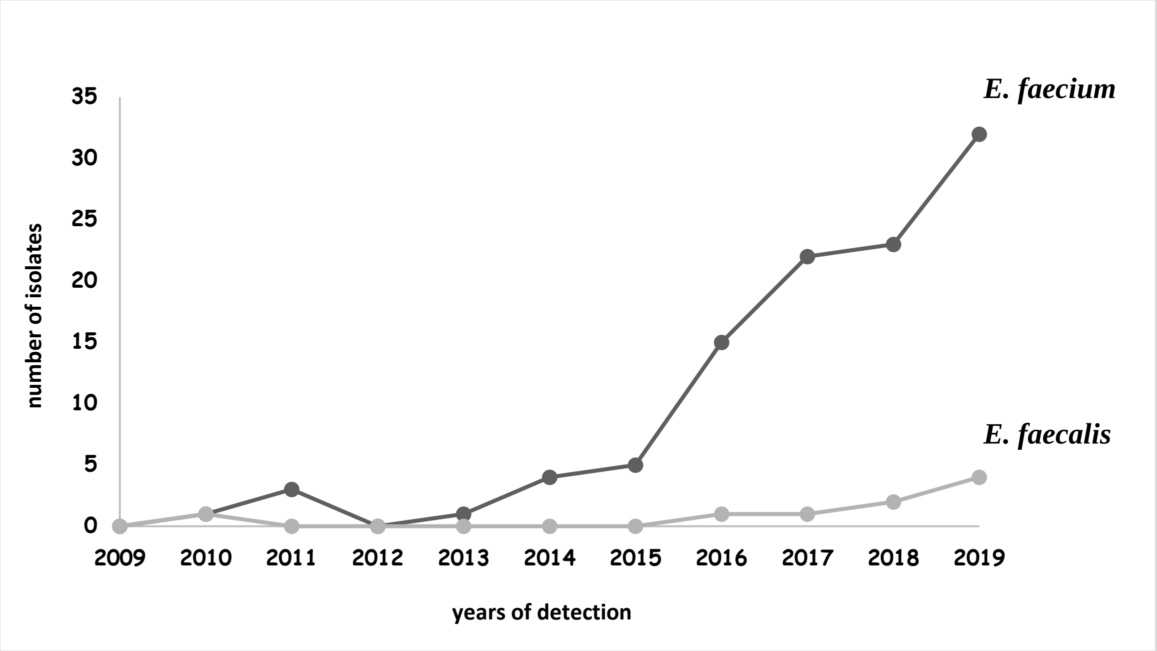

Figure S1: Detection of linezolid resistant enterococci in the Czech Republic from 2009 to 2019. Increasing pattern of linezolid resistant enterococci has been observed in this study: *E. faecium*: 2009/0; 2010/1; 2011/3; 2012/0; 2013/1; 2014/4; 2015/5; 2016/15; 2017/22; 2018/23; 2019/32, *E. faecalis*: 2009/0; 2010/1; 2011/0; 2012/0; 2013/0; 2014/0; 2015/0; 2016/1; 2017/1; 2018/2; 2019/4.

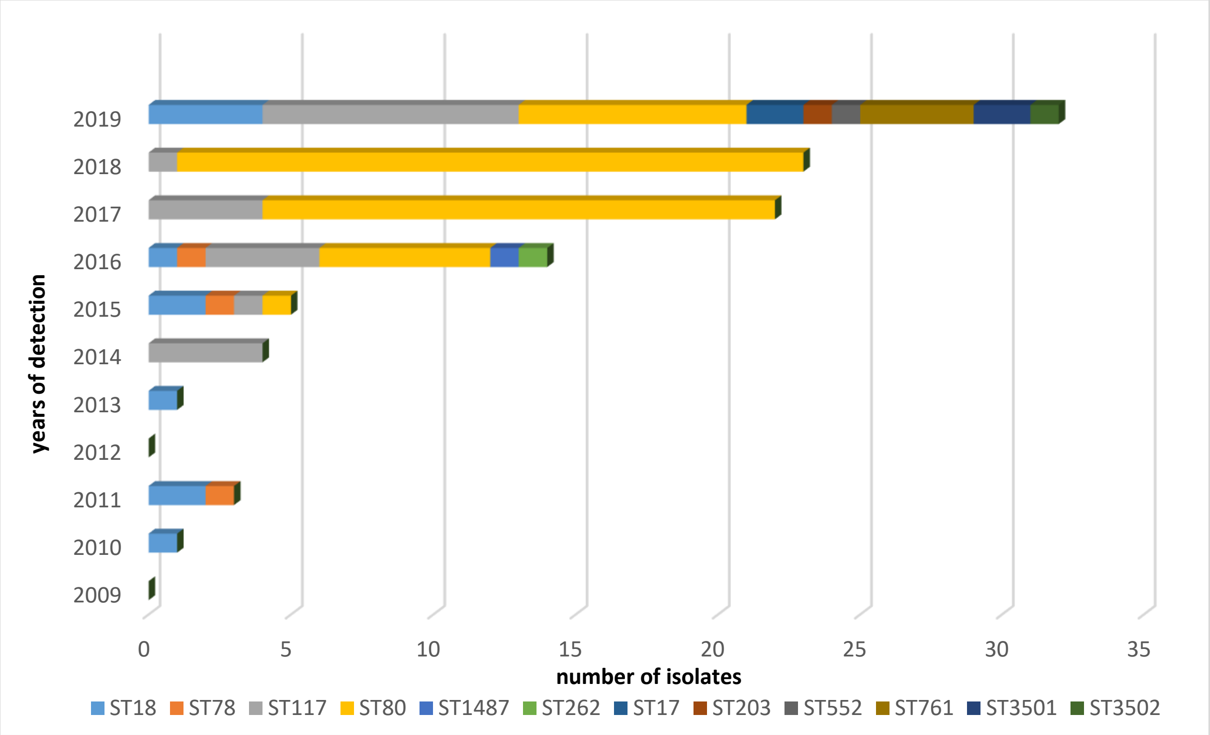

Figure S2: STs occurrence in the group of linezolid resistant *E. faecium* (n=106) strains from 2009 to 2019. STs detected in the study were: ST80 (n=53), ST117 (n=24), ST18 (n=13), ST761 (n=4), ST78 (n=3), ST17 (n=2), ST203 (n=1), ST552 (n=1), ST262 (n=1), ST1487 (n=1), ST3501 (n=2), ST3502 (n=1).

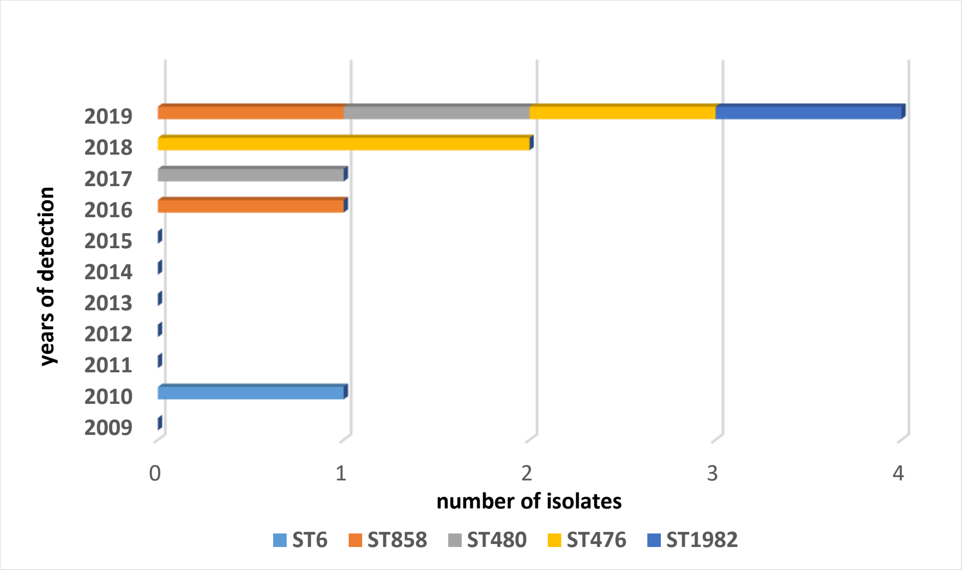

Figure S3: STs occurrence in the group of linezolid resistant *E. faecalis* (n=9) isolates from 2009 to 2019. STs detected in the study were ST6 (n=1), ST476 (n=3), ST480 (n=2), ST858 (n=2), ST1982 (n=1).

Supplementary data

Table S1. Phenotypic and genotypic characteristics of the 115 linezolid-resistant enterococcal isolates examined in the NRL for ATB between 2009 and 2019.

Isolates	Isolation year	Clinical specimen	Sex	Hospital	Age (years)	MIC(mg/L)							mechanism of LNZ resistance	*van* genotype	ST/CC
						AMP	LNZ	TEI	VAN	GEN	STR	TGC			
E. faecium															
**4 088**	2010	swab	F	Brno	43	> 64	>16	0.5	> 32	>256	>1024	0.06	ΔG2576T	*vanA*, *vanB*	18/ CC17
**9 954**	2011	urine	F	Prague	36	> 64	16	>32	> 32	<64	1024	0.06	ΔG2576T	*vanA*, *vanB*	78/ CC17
**9 996**	2011	catheter	M	Plzen	61	> 64	16	>32	> 32	<64	1024	0.06	ΔG2576T	*vanA*, *vanB*	18/ CC17
**10 002**	2011	swab	F	Brno	20	> 64	>16	32	> 32	>256	>1024	0.06	ΔG2576T	*vanA*	18/ CC17
**22 839**	2013	fistula - swab	F	Plzen	25	> 64	16	>32	> 32	>256	<256	0.06	ΔG2576T	*vanA*	18/ CC17
**25 031**	2014	throat swab	M	Plzen	67	> 64	16	< 0.25	> 32	256	>1024	0.125	ΔG2576T	*vanB*	117/ CC17
**25 100**	2014	rectal swab	M	Plzen	38	> 64	>16	< 0.25	> 32	256	>1024	0.125	ΔG2576T	*vanB*	117/ CC17
**25 265**	2014	urine	F	Ceske Budejovice	69	> 64	16	32	> 32	256	>1024	0.06	ND	*vanA*	117/ CC17
**25 298**	2014	rectal swab	M	Plzen	61	> 64	16	16	> 32	256	>1024	<0.03	ΔG2576T	*vanA*	117/ CC17
**28 182**	2015	rectal swab	F	Brno	67	> 64	>16	> 32	> 32	>256	>1024	<0.03	ΔG2576T	*vanA*	18/ CC17
**28 601**	2015	swab	M	Brno	38	> 64	>16	< 0.25	0.5	>256	>1024	0.06	ΔG2576T	-	117/ CC17
**29 050**	2015	secrete	M	Brno	66	> 64	16	< 0.25	0.5	>256	<256	0.125	ΔG2576T	-	78/ CC17
**29 126**	2015	rectal swab	F	Brno	49	> 64	>16	>32	> 32	<64	<256	< 0.03	ΔG2576T	*vanA*	18/ CC17
**32 103**	2015	blood	F	Brno	85	> 64	8	32	> 32	>256	>1024	< 0.03	ΔG2576T	*vanA*	80/ CC17
**32 951**	2016	swab	M	Brno	76	> 64	>16	32	> 32	>256	>1024	< 0.03	ΔG2576T	*vanA*	80/ CC17
**32 960**	2016	secrete	F	Brno	63	> 64	>16	0.5	1	>256	>1024	0.06	ΔG2576T	-	117/ CC17
**33 234**	2016	throat swab	M	Brno	69	> 64	>16	> 32	> 32	>256	>1024	< 0.03	ΔG2576T	*vanA*	80/ CC17

**33 269**	2016	blood	M	Brno	59	> 64	16	< 0.25	0.5	>256	1024	0.06	ΔG2576T	-	117/ CC17
**33 992**	2016	rectal swab	F	Brno	29	> 64	>16	32	> 32	>256	<256	0.06	ΔG2576T	*vanA*	18/ CC17
**34 237**	2016	swab	F	Brno	77	> 64	>16	32	> 32	>256	>1024	0.06	ΔG2576T	*vanA*	80/ CC17
**34 484**	2016	throat swab	unkno wn	Plzen	54	> 64	16	> 32	> 32	>256	>1024	0.06	ΔG2576T	*vanA*	117/ CC17
**34 605**	2016	urine	M	Brno	81	> 64	16	32	> 32	>256	>1024	0.06	ΔG2576T	*vanA*	80/ CC17
**35 041**	2016	punctate	M	Brno	52	> 64	>16	< 0.25	1	>256	>1024	< 0.03	ΔG2576T	-	117/ CC17
**35 042**	2016	rectal swab	M	Brno	40	> 64	8	> 32	> 32	>256	>1024	0.06	*cfr*	*vanA*	117/ CC17
**35 262**	2016	rectal swab	F	Brno	76	> 64	>16	32	> 32	>256	>1024	0.06	ΔG2576T	*vanA*	80/ CC17
**35 263**	2016	urine	M	Brno	82	> 64	>16	32	> 32	>256	>1024	0.06	ΔG2576T	*vanA*	80/ CC17
**35 548**	2016	swab	M	Usti nad Labem	62	> 64	>16	> 32	> 32	>256	>1024	0.06	ND	*vanA*	1487/ CC17
**36 574**	2016	blood	M	Prague	59	> 64	8	< 0.25	0.5	<64	>1024	0.06	ΔG2576T	-	78/ CC17
**37 310**	2016	surgical wound	F	Prague	78	> 64	8	< 0.25	0.5	<64	>1024	0.125	*optrA*	-	262/ CC17
**37 809**	2017	swab	M	Brno	73	> 64	>16	32	> 32	>256	>1024	0.06	ΔG2576T	*vanA*	80/ CC17
**38 243**	2017	aspirate	M	Nové Město na Moravě	65	> 64	16	32	> 32	>256	>1024	< 0.03	ΔG2576T	*vanA*	80/ CC17
**39 203**	2017	rectal swab	F	Brno	51	> 64	16	32	> 32	>256	>1024	0.06	ΔG2576T	*vanA*	80/ CC17
**39 403**	2017	throat swab	F	Brno	62	> 64	16	32	> 32	>256	<256	0.06	ΔG2576T	*vanA*	80/ CC17
**39 549**	2017	rectal swab	M	Brno	18	> 64	>16	32	> 32	>256	>1024	0.06	ΔG2576T	*vanA*	80/ CC17
**39 821**	2017	rectal swab	M	Brno	68	> 64	>16	32	> 32	>256	>1024	0.125	ΔG2576T	*vanA*	80/ CC17
**39 822**	2017	rectal swab	M	Brno	71	> 64	>16	32	> 32	>256	>1024	0.065	ΔG2576T	*vanA*	117/ CC17
**39 823**	2017	rectal swab	F	Brno	63	> 64	>16	32	> 32	>256	>1024	0.125	ΔG2576T	*vanA*	80/ CC17
**40 238**	2017	mouth swab	M	Brno	65	> 64	>16	> 32	> 32	>256	>1024	0.125	ΔG2576T	*vanA*	80/ CC17
**40 239**	2017	rectal swab	F	Brno	73	> 64	>16	> 32	> 32	< 64	>1024	0.125	ΔG2576T	*vanA*	80/ CC17
**40 536**	2017	urine	F	Brno	42	> 64	16	> 32	> 32	>256	>1024	0.06	ΔG2576T	*vanA*	117/ CC17
**40 537**	2017	rectal swab	M	Brno	45	> 64	>16	> 32	> 32	>256	>1024	0.125	ΔG2576T	*vanA*	80/ CC17
**41 094**	2017	rectal swab	F	Brno	40	> 64	>16	> 32	> 32	>256	>1024	0.125	ΔG2576T	*vanA*	117/ CC17

**41 178**	2017	blood	M	Prague	47	> 64	>16	< 0.25	1	>256	>1024	0.125	ΔG2576T	-	117/ CC17
**41 694**	2017	rectal swab	F	Ostrava	71	> 64	>16	32	> 32	>256	>1024	0.06	ΔG2576T	*vanA*	80/ CC17
**41 942**	2017	rectal swab	F	Brno	50	> 64	16	32	> 32	>256	>1024	0.06	ΔG2576T	*vanA*	80/ CC17
**41 943**	2017	swab	M	Brno	68	> 64	16	32	> 32	>256	<256	0.06	ΔG2576T	*vanA*	80/ CC17
**42 042**	2017	rectal swab	M	Brno	58	> 64	>16	32	> 32	>256	>1024	0.06	ΔG2576T	*vanA*	80/ CC17
**42 043**	2017	rectal swab	F	Brno	44	> 64	>16	> 32	> 32	>256	>1024	0.06	ΔG2576T	*vanA*	80/ CC17
**42 238**	2017	rectal swab	M	Brno	64	> 64	>16	> 32	> 32	>256	>1024	0.125	ΔG2576T	*vanA*	80/ CC17
**42 239**	2017	rectal swab	F	Brno	48	> 64	>16	> 32	> 32	>256	>1024	0.06	ΔG2576T	*vanA*	80/ CC17
**42 240**	2017	rectal swab	unknown	Brno	52	> 64	>16	32	> 32	>256	>1024	0.06	ΔG2576T	*vanA*	80/ CC17
**42 367**	2018	rectal swab	F	Brno	22	> 64	>16	32	> 32	>256	>1024	0.125	ΔG2576T	*vanA*	80/ CC17
**42 368**	2018	rectal swab	F	Brno	53	> 64	>16	> 32	> 32	>256	>1024	0.125	ΔG2576T	*vanA*	80/ CC17
**42 523**	2018	swab	F	Brno	68	> 64	>16	32	> 32	>256	>1024	0.125	ΔG2576T	*vanA*	80/ CC17
**42 750**	2018	catheter	M	Brno	64	> 64	>16	> 32	> 32	>256	>1024	0.125	ΔG2576T	*vanA*	80/ CC17
**42 943**	2018	stool sample	F	Brno	58	> 64	>16	> 32	> 32	>256	>1024	0.06	ΔG2576T	*vanA*	80/ CC17
**42 944**	2018	swab	M	Brno	64	> 64	>16	> 32	> 32	>256	>1024	0.06	ΔG2576T	*vanA*	80/ CC17
**43 190**	2018	secrete	M	Brno	64	> 64	>16	0.5	1	>256	<256	0.125	ΔG2576T	-	80/ CC17
**43 303**	2018	rectal swab	M	Brno	63	> 64	>16	> 32	> 32	>256	>1024	0.125	ΔG2576T	*vanA*	80/ CC17
**43 412**	2018	ascites	F	Brno	72	> 64	>16	32	> 32	>256	>1024	0.125	ΔG2576T	*vanA*	80/ CC17
**43 651**	2018	mouth swab	F	Brno	81	> 64	>16	> 32	> 32	>256	>1024	0.125	ΔG2576T	*vanA*	80/ CC17
**43 714**	2018	swab	M	Brno	79	> 64	>16	32	> 32	>256	>1024	0.125	ΔG2576T	*vanA*	80/ CC17
**44 267**	2018	urine	M	Ostrava	83	> 64	>16	> 32	> 32	>256	>1024	0.125	ΔG2576T	*vanA*	80/ CC17
**44 467**	2018	swab	M	Brno	72	> 64	16	> 32	> 32	>256	>1024	0.125	ΔG2576T	*vanA*	80/ CC17
**45 511**	2018	rectal swab	M	Brno	63	> 64	16	> 32	> 32	>256	<256	0.125	ΔG2576T	*vanA*	80/ CC17
**45 512**	2018	mouth swab	M	Brno	63	> 64	>16	0.5	0.5	>256	>1024	0.06	ΔG2576T	-	80/ CC17
**45 690**	2018	rectal swab	F	Brno	64	> 64	>16	> 32	> 32	>256	<256	0.125	ΔG2576T	*vanA*	80/ CC17

**45 990**	2018	bile	M	Hradec Králové	61	> 64	8	> 32	> 32	>256	>1024	0.125	ΔG2576T	*vanA*	80/ CC17
**46 153**	2018	sputum	M	Brno	87	> 64	>16	0.5	1	>256	<256	0.125	ΔG2576T	-	18/ CC17
**46 334**	2018	sputum	M	Hradec Králové	60	> 64	8	0.5	1	>256	>1024	0.06	ΔG2576T	-	117/CC17
**46 347**	2018	swab	F	Brno	41	> 64	16	0.5	1	>256	<256	0.125	ΔG2576T	-	18/CC17
**46 586**	2018	urine	F	Brno	60	> 64	16	> 32	> 32	>256	>1024	0.125	ΔG2576T	*vanA*	80/ CC17
**47 053**	2018	rectal swab	M	Brno	61	> 64	>16	> 32	> 32	>256	>1024	0.125	ΔG2576T	*vanA*	80/ CC17
**47 267**	2018	urine	F	Brno	64	> 64	>16	32	> 32	>256	<256	0.06	ΔG2576T	*vanA*	80/ CC17
**48 643**	2019	tracheal aspirate	M	Hradec Králové	65	> 64	8	< 0.25	1	<64	>1024	0.125	ΔG2576T	-	117/ CC17
**49 053**	2019	rectal swab	M	Hradec Králové	64	> 64	8	< 0.25	0.5	<64	<256	0.06	ΔG2576T	-	117/ CC17
**49 083**	2019	swab	F	Brno	77	> 64	>16	0.5	1	>256	<256	0.125	ΔG2576T	-	18/CC17
**49 226**	2019	stool sample	F	Brno	69	> 64	>16	> 32	> 32	>256	>1024	0.125	ΔG2576T	*vanA*	80 CC17
**49 642**	2019	bronchoalveolar lavage	F	Brno	84	> 64	>16	< 0.25	0.5	>256	<256	0.125	ΔG2576T	-	18/ CC17
**49 853**	2019	swab	M	Brno	56	> 64	16	< 0.25	1	>256	>1024	0.125	ΔG2576T	-	18/ CC17
**50 313**	2019	catheter	M	Brno	72	> 64	>16	0.5	1	>256	<256	0.125	ΔG2576T	-	18/ CC17
**50 678**	2019	blood	M	Brno	77	> 64	>16	> 32	> 32	>256	<256	0.125	ΔG2576T	*vanA*	3501*/none
**50 679**	2019	secrete	M	Brno	25	> 64	16	< 0.25	0.5	<64	>1024	0.06	ΔG2576T	-	117/ CC17
**50 955**	2019	rectal swab	M	Brno	55	> 64	16	> 32	> 32	>256	<256	1	ΔG2576T	*vanA*	80/ CC17
**50 956**	2019	rectal swab	M	Brno	71	> 64	16	> 32	> 32	>256	<256	0.06	ΔG2576T	*vanA*	3501*/none
**50 962**	2019	aspirate	M	Hradec Králové	60	> 64	8	> 32	> 32	>256	>1024	0.06	ΔG2576T	*vanA*	80/ CC17
**51 138**	2019	swab	M	Klatovy	60	> 64	16	> 32	> 32	>256	>1024	0.25	ΔG2576T	*vanA*	80/ CC17
**51 324**	2019	blood	M	Plzen	46	> 64	16	> 32	> 32	<64	>1024	0.125	ΔG2576T	*vanA*	203/ CC17
**51 498**	2019	throat swab	M	Plzen	86	> 64	16	> 32	0.5	>256	<256	0.125	ΔG2576T	-	552/ CC17
**51 702**	2019	rectal swab	F	Brno	64	> 64	>16	32	> 32	>256	<256	0.06	ΔG2576T	*vanA*	117/ CC17
**51 703**	2019	rectal swab	F	Brno	70	> 64	>16	>32	> 32	>256	<256	0.05	ΔG2576T	*vanA*	80/ CC17
**51 981**	2019	rectal swab	M	Brno	82	> 64	>16	32	> 32	<64	<256	0.06	ΔG2576T	*vanA*	117/ CC17

**51 982**	2019	abscess swab	M	Brno	80	> 64	16	0.5	0.5	>256	>1024	0.06	ND	-	761/ CC17
**52 074**	2019	rectal swab	M	Hradec Králové	46	> 64	16	0.5	0.5	<64	>1024	0.125	ΔG2576T	-	117/ CC17
**52 155**	2019	stool sample	M	Hradec Králové	78	> 64	16	> 32	> 32	>256	>1024	0.06	ΔG2576T	*vanA*	80/ CC17
**52 187**	2019	rectal swab	F	Brno	28	> 64	16	32	> 32	>256	>1024	0.06	ΔG2576T	*vanA*	117/ CC17
**52 188**	2019	swab	M	Brno	74	> 64	8	32	> 32	>256	>1024	0.06	ΔG2576T	*vanA*	761/ CC17
**52 189**	2019	swab	M	Brno	25	> 64	16	< 0.25	1	>256	<256	0.125	ΔG2576T	-	80/ CC17
**52 493**	2019	rectal swab	M	Brno	41	> 64	>16	> 32	> 32	<64	>1024	0.06	ΔG2576T	*vanA*, *vanB*	117/ CC17
**52 739**	2019	bile	F	Brno	50	> 64	>16	< 0.25	1	<64	<256	0.125	ΔG2576T	-	17/ CC17
**52 740**	2019	rectal swab	F	Brno	43	> 64	>16	> 32	> 32	>256	<256	0.5	ΔG2576T	*vanA*	80/ CC17
**52 794**	2019	unknown	M	Hradec Králové	63	> 64	16	0.5	0.5	>256	>1024	0.06	ΔG2576T	-	761/ CC17
**52 949**	2019	swab	F	Brno	50	> 64	>16	32	> 32	<64	<256	0.125	ΔG2576T	*vanA*	117/ CC17
**53 213**	2019	rectal swab	F	Hradec Králové	78	> 64	8	< 0.25	0.5	>256	1024	0.06	ΔG2576T	-	117/ CC17
**54 389**	2019	sputum	M	Brno	26	> 64	16	< 0.25	0.5	<64	<64	0.06	ΔG2576T	-	761/ CC17
**54 585**	2019	rectal swab	M	Prague	50	4	8	< 0.25	0.5	<64	>1024	0.125	*optrA*	-	3502*/ none
E. faecalis															
**4 672**	2010	blood	M	Brno	63	<0.5	>16	< 0.25	1	>256	>1024	<0.03	ΔG2576T	-	6/CC2
**37 203**	2016	urine	M	Prague	43	1	8	< 0.25	1	>256	>1024	0.06	*optrA*	-	858/none
**40 812**	2017	swab	M	Brno	66	1	8	< 0.25	1	>256	<256	0.06	*optrA*	-	480/CC480
**46 152**	2018	wound swab	F	Brno	49	1	8	< 0.25	1	>256	<256	0.125	*optrA*	-	476/CC476
**48 089**	2018	swab	F	Ostrava	69	1	8	< 0.25	1	>256	<256	0.25	*optrA*	-	476/CC476
**49 852**	2019	urethral swab	M	Brno	66	1	8	< 0.25	1	>256	>1024	0.125	*optrA*	-	480/CC480
**50 460**	2019	wound swab	M	Prague	50	1	8	< 0.25	1	>256	>1024	0.125	*optrA*	-	858/none
**50 875**	2019	urethral swab	M	Novy Jicin	44	1	8	< 0.25	1	>256	<256	0.125	*optrA*	-	476/CC476
**54 695**	2019	urine	F	Prague	55	1	16	< 0.25	1	>256	<256	0.06	ΔG2576T	-	1982/ none

Abbreviations: MIC, minimal inhibitory concentration; AMP, ampicillin; LNZ, linezolid; TEI, teicoplanin; VAN, vancomycin; GEN, gentamicin; STR, streptomycin; TGC, tigecycline; -, wild type, ND, non-detected mechanism; ST, sequence type; CC, clonal complex; *, novel sequencing type

Table S2. Primers used in the study.

Primer purpose	Amplified gene	Primer pair	Nucleotide Sequence (5'-3')	PCR conditions
**PCR to confirm mechanism of linezolid-resistance**	*cfr*	*cfr*-F<br>*cfr*-R	TGAAGTATAAAGCAGGTTGGGAGTCA<br>ACCATATAATTGACCACAAGCAGC	94ºC for 3 min. 36 cycles of 94ºC for 45 seconds, 61ºC for 45 seconds, 72ºC for 1 min. Final elongation 10 min at 72ºC
	cfr_B	*cfr_B*-F<br>*cfr_B*-R	AGCTGGTTGGGAGTCATTT<br>TCGCTAATGCTTCTCCCAT	94ºC for 3 min. 36 cycles of 94ºC for 45 seconds, 51ºC for 45 seconds, 72ºC for 1 min. Final elongation 10 min at 72ºC
	poxtA	*poxtA*-F<br>*poxtA*-R	TCAATGCAGAGCAGGAAGCA<br>GGTGGATTTACCGACACCGT	94ºC for 3 min. 36 cycles of 94ºC for 45 seconds, 50ºC for 45 seconds, 72ºC for 1 min. Final elongation 10 min at 72ºC
	optrA	*optrA*-F<br>*optrA*-R	CAGGTGGTCAGCGAACTAAG<br>GCCACACCACCCATAAGTGTT	94ºC for 3 min. 35 cycles of 94ºC for 1 min, 59ºC for 45 seconds, 72ºC for 2 min. Final elongation 10 min at 72ºC
	L3	*rrlC*-F<br>*rrlC*-R	GCGCTTCATTCGTGAATTCAA<br>TTCTTTCTGCATCGACACGTACAA	94ºC for 5 min. 36 cycles of 94ºC for 30 seconds, 50ºC for 30 seconds, 72ºC for 1 min. Final elongation 10 min at 72ºC
	L4	*rrlD*-F<br>*rrlD*-R	ACGATGCAATCGTAATGCAA<br>TTCAGCAACTTTTTCTGACAA	94ºC for 3 min. 35 cycles of 94ºC for 1 min, 51ºC for 45 seconds, 72ºC for 2 min. Final elongation 10 min at 72ºC
	L22	*rplV*-F<br>*rplV*-R	GGACATGCTGCTGACGATA<br>ACCATTTAGCATCCCAGTCG	94ºC for 5 min. 36 cycles of 94ºC for 30 seconds, 50ºC for 30 seconds, 72ºC for 1 min. Final elongation 10 min at 72ºC
	23S rDNA	*23S* rDNA-F<br>*23S* rDNA-F	GTAACGATTTGGGCACTGTCG<br>CGATTAGTATTGGTCCGCTC	94ºC for 1 min. 33 cycles of 94ºC for 30 seconds, 55ºC for 30 seconds, 72ºC for 7 min. Final elongation 10 min at 72ºC
**PCR to confirm *van* gene type**	*vanA*	*vanA*-F<br>*vanA*-R	GGGAAAACGACAATTGC<br>GTACAATGCGGCCGTTA	94ºC for 2 min. 31 cycles of 94ºC for 1 min, 54ºC for 1 min, 72ºC for 1 min. Final elongation 10 min at 72ºC
	vanB	*VanB*-F<br>*VanB*-R	ATGGGAAGCCGATAGTC<br>GATTTCGTTCCTCGACC	

Table S3. Primers used in the MLST analysis of *E. faecium* and *E. faecalis* isolates.

Amplified gene	Primer pair	Nucleotide Sequence (5'-3')	PCR conditions
E. faecium			
adk	*adk-F*<br>*adk-R*	GAACCTCATTTTAATGGGG<br>TGATGTTGATAGCCAGACG	94ºC for 15 min. 35 cycles of 94ºC for 30s, 50ºC for 30s 72ºC for 30s. Final elongation 5 min at 72ºC
atpA	*atpA-F*<br>*atpA-R*	TTCAAATGGCTCATACGG<br>AGTTCACGATAAGCAACAGC	
ddl	*ddl-F*<br>*ddl-R*	GAGACATTGAATATGCCTTATG<br>AAAAAGAAATCGCACCG	
gyd	*gyd-F*<br>*gyd-R*	CAAACTGCTTAGCTCCAATGGC<br>CATTTCGTTGTCATACCAAGC	
gdh	*gdh-F*<br>*gdh-R*	GGCGCACTAAAAGATATGGT<br>CCAAGATTGGGCAACTTCGTCCCA	
purK	*purK-F*<br>*purK-R*	CAGATTGGCACATTGAAAG<br>TTCATTCACATATAGCCCG	
pstS	*pstS-F*<br>*pstS-R*	TTGAGCCAAGTCGAAGCTGGAG<br>CGTGATCACGTTCTACTTCC	
E. faecalis			94ºC for 5 min. 30 cycles of 94ºC for 30s, 52ºC for 30s 72ºC for 1minute. Final elongation 7 min at 72ºC
gdh	*gdh-F*<br>*gdh-R*	GGCGCACTAAAAGATATGGT<br>CCAAGATTGGGCAACTTCGTCCCA	
gyd	*gyd-F*<br>*gyd-R*	CAAACTGCTTAGCTCCAATGGC<br>CATTTCGTTGTCATACCAAGC	
pstS	*pstS-F*<br>*pstS-R*	CGGAACAGGACTTTCGC<br>ATTTACATCACGTTCTACTTGC	
gki	*gki-F*<br>*gki-R*	GATTTTGTGGGAATTGGTATGG<br>ACCATTAAAGCAAAATGATCGC	
aroE	*aroE-F*<br>*aroE-R*	TGGAAAACTTTACGGAGACAGC<br>GTCCTGTCCATTGTTCAAAAGC	
xpt	*xpt-F*<br>*xpt-R*	AAAATGATGGCCGTGTATTAGG<br>AACGTCACCGTTCCTTCACTTA	
yiqL	*yiqL-F*<br>*yiqL-R*	CAGCTTAAGTCAAGTAAGTGCCG<br>GAATATCCCTTCTGCTTGTGCT	
